# Breeding of a thermostable xylanase-producing strain of *Myceliophthora thermophila* by atmospheric room temperature plasma (ARTP) mutagenesis

**DOI:** 10.3389/fbioe.2022.1095323

**Published:** 2023-01-05

**Authors:** Ning Zhang, Yue Jiang, Yun-Juan Sun, Jian-Chun Jiang, Ya-Juan Tong

**Affiliations:** ^1^ Key Lab of Biomass Energy and Material, Key Lab of Chemical Engineering of Forest Products, National Engineering Research Center of Low-Carbon Processing and Utilization of Forest Biomass, Jiangsu Co-Innovation Center of Efficient Processing and Utilization of Forest Resource, National Forestry and Grassland Administration, Institute of Chemical Industry of Forest Products, Chinese Academy of Forestry (CAF), Nanjing, Jiangsu, China; ^2^ Co-Innovation Center of Efficient Processing and Utilization of Forest Resources, Nanjing Forestry University, Nanjing, China

**Keywords:** xylanase, *Myceliophthora thermophila*, thermotolerant mutant, antioxidase, ARTP

## Abstract

**Introduction:** Hemicellulose is an important component in lignocellulose materials, which is second only to cellulose, accounting for 15%–35% of the dry weight of plants. In the current situation of energy shortage, making full use of lignocellulose materials to produce fuel ethanol has become an important way to solve the energy problem. Xylanase plays a crucial role in the utilization of hemicellulose. It is a necessary means to reduce the cost of hemicellulose utilization by improving the activity of xylanase. Moreover, most naturally xylanases are mesophilic enzymes, which limits their industrial application.

**Methods:**In this study, *Myceliophthora thermophila* was used to produce xylanases and a thermostable mutant *M* 2103 was obtained by atmospheric room temperature plasma (ARTP) mutagenesis. The research work started with exploring the effects of ARTP mutagenesis on the antioxidase system [superoxide dismutase (SOD), catalase (CAT), peroxidase (POD), polyphenol oxidase (PPO), and antioxidant capacity (AOC)] of *M. thermophile*, and found that superoxide dismutase activity increased by 221.13%, and polyphenol oxidase activity increased by 486.04% as compared with the original strain when the implantation time was 300 s. So as to determine the conditions for subsequent mutagenesis.

**Results and Discussion:**For the mutant *M* 2103, the reaction temperature for xylanase production remained stable in the range of 70°C–85°C. Its optimum temperature was 75°C, which was 15°C higher than that of the original strain. And its xylanase activity increased by 21.71% as compared with the original strain. *M* 2103 displayed a significantly higher relative xylanase activity than the original strain in the acidic (pH 4.0–7.0) range, and the xylanase activity was relatively stable in the pH range of 6.0–8.5. These results provide an alternative biocatalyst for the production of xylooligosaccharide, and a potential usage of ARTP in the mutagenesis of thermostable mutant.

## Introduction

Xylan is one of the major components of the plant cell wall. It is the second richest, renewable resource in nature after cellulose (Biely et al., 2016). The structure of xylan is very complex. Its main chain is formed by the polymerization of d-xylopyranose through a β-1, 4-glycosidic bond, and the side chain carries an acetyl group, galactose, arabinose, glucuronic acid and other substituent groups. The degradation of xylan requires the joint action of an enzyme system composed of variety of enzymes. Among them, xylanase (EC 3.2.1.8) is the key enzyme, which hydrolyzes β-1, 4-glycosidic bonds from the interior of the main chain to produce a mass of xylo-oligosaccharides and a small amount of xylose (Csiszár et al., 2015; Xu et al., 2016). Xylanase has many applications, including baking (Wu et al., 2018), fruit juice processing (Bajaj and Manhas, 2012), brewing (Wang et al., 2016), feed processing, papermaking and biofuels (Wang et al., 2013; Le et al., 2018). In the current situation of energy shortage, xylanase plays an important role in the process of preparing fuel ethanol from lignocellulosic materials. It can promote the degradation of lignocellulosic materials, improve the production of monosaccharides glucose, and then improve the ethanol yield in the next step of fermentation. On the other side, xylanase is used to directly hydrolyze xylan into xylose, and then the xylose is used to produce fuel ethanol by the special bacteria or yeast.

Among the current pretreatment methods for fuel ethanol production, the acidic electrolytiyed water (Shen et al., 2014) (AEW) is pretreated at high temperature (above 140°C) and strong acid (pH 2.0–2.2). Since the pH value of high pressure hot water (above 200°C) is 5.6, liquid hot water (Saksit et al., 2021) (LHW) pretreatment is also a kind of dilute acid pretreatment. In addition, the simultaneous saccharification and fermentation (Suryawati et al., 2009) (SSF) of lignocellulosic materials to produce fuel ethanol is also carried out under the condition of high temperature (40°C–50°C) and weak acid (pH 4.0–5.0). Therefore, it is particularly important to screen and construct the high temperature and acid resistant xylanase.

Thermostable xylanase is a product of numerous thermophilic microorganisms, including *Thermomyces lanuginosus* (Wang et al., 2012), *Thermophilic sporotrichum* (Ghosh et al., 2019), *Paecilomyces thermophila* (Zhang et al., 2010), *Myceliophthera thermophila* (Dahiya and Singh, 2019), *Thermosaccharolyticum* (Jiang et al., 2017) and other thermophilic fungi, as well as thermophilic bacteria (Saleem et al., 2009) and thermophilic archaea (Knapik et al., 2019). It has been reported that bacteria can usually produce low molecular weight alkaline xylanase and high molecular weight acid xylanase, while fungi usually produce only high molecular weight acid xylanase. *M. thermophila* is classified as a thermophilic fungus that produces a variety of thermostable enzymes including cellulase (Fan et al., 2015), pectinase (Hollmann et al., 2016), esterase (Kool et al., 2014), phytase (Seema et al., 2020) and endoxylanase (Gool et al., 2012). Xylanase from *M. thermophila* has been purified and its properties have been studied (Boonrung et al., 2016). It has significant endonuclease β-1,4-xylanase activity without exoxylanase activity (Vafiadi et al., 2010) and it can be used to produce xylooligosaccharides.

At present, there are two primary strategies that can be employed to improve the thermal stability of xylanase. One involves directly screening thermophilic microbial strains from a high temperature environment, and the other involves modifying xylanase *via* genetic engineering technology. Since the end of the 20th century, numerous xylanase gene sequences have been cloned, and their enzymatic properties were determined. However, there are few microorganisms able to produce thermostable xylanase, and the obtained strains usually have low enzymatic activity and harsh culture conditions that make it challenging to meet the demands of industrial production (Zhang et al., 2014a; Ghosh et al., 2019). Therefore, it is imperative to use modern bioengineering technology to genetically improve xylanase-producing strains.

As a new physical mutation method, ARTP technology was developed by Xing Xinhui’s team of Tsinghua University based on the radio-frequency atmospheric pressure glow discharge (RF APGD), which was a gentle RF APGD and can be applied in biotechnology research. The operation parameters, including RF power input, helium flow rate, plasma range and processing time, are controlled by programmable controller to make the mutagenesis of microorganisms more efficient and faster. Studies have shown that ARTP can react with biological macromolecules such as DNA (Wang et al., 2020) and protein in biological cells (Zhang et al., 2014b), resulting in lethal or sublethal effects. There are a large number of active oxides, active nitrides and neutral active particles in ARTP, including superoxide anion (O^2-^), hydroxyl radical (-OH), ozone (O_3_), hydroperoxide anion (HO^2-^), singlet oxygen (^1^O_2_), alkoxy (RO^−^), peroxynitroso anion (ONOO^−^) and so on. Among them, active oxides and active nitrides are the key factors for ARTP to damage DNA double-stranded structure and induce DNA damage repair (DDR) response or SOS response. These active substances will act on the O-H bond, O-P bond and N-C glycosidic bond in the DNA chain structure to damage the molecular structure of DNA, and then make DNA single strand break. When the break sites on different single strands are close, DNA double strand break will occur. Therefore, ARTP can cause a wider range of gene mutations. Moreover, at atmospheric pressure, ARTP temperature can be controlled in the room temperature range, suitable for biological treatment (Zhang et al., 2014a). At present, ARTP has been successfully applied in mutation breeding of more than 40 kinds of microorganisms, including bacteria, fungi and microalgae, which can change the genetic material, protein structure, metabolic level and other factors of microorganisms to improve the production of microbial metabolites. And it has increasingly attracted attention in the field of microbial mutation breeding and biomedicine and has become a considerably active interdisciplinary research field (Li et al., 2015; Cheng et al., 2016; Gu et al., 2017).

The previous studies (Finkel, 2003) have shown that the activities of antioxidant enzymes in microorganisms was sensitive to the ion implantation, and the changes of antioxidant enzymes was also one of various internal factors, which influence their lives activity. So this work started with exploring the effects of ARTP mutagenesis on the antioxidase system of *M. thermophile*, and determined the best mutagenic conditions. For the mutant *M* 2103, its xylanase properties including the optimum pH and optimum reaction temperature were tested.

## Materials and methods

### Strains

*M. thermophila* was purchased from the China Center of Industrial Culture Collection (CICC), and numbers for CICC 2441. It was an aerobic microorganism and its culture temperature was 45°C.

### Medium

Isolation medium (g/L): glucose 20.00, KH_2_PO_4_ 3.00, MgSO_4_.7H_2_O 1.50, (NH_4_)_2_SO_4_ .75, wheat bran decoction (Cavalcante et al., 2008) (100 g of bran, 900 g of water, boiling for 20 min, filtered through four layers of gauze) 80.00, agar 15.00. Seed medium (g/L): malt extract 30.00, soya peptone 3.00. Xylanase fermentation medium (g/L): glucose 20.00, xylan .1, KH_2_PO_4_ 3.00, MgSO_4_.7H_2_O 1.50, (NH_4_)_2_SO_4_ .75, wheat bran decoction 80.00. All mediums were sterilized at 121°C for 20 min.

## Breeding of *M. thermophila via* ARTP

### ARTP mutagenesis method

ARTP mutagenesis was performed with the ARTP Mutation Breeding System (Wuxi TMAXTREE Biotechnology Co. Ltd) (Wuxi, Jiangsu, China). *M. thermophila* CICC 2441 was used as the original strain which was purchased from the China Center of Industrial Culture Collection (CICC). Before mutagenesis, *M. thermophila* CICC 2441 was cultured in the isolation medium for 5 days at 45°C. The spores were scraped off with 10 ml of normal saline and transferred to a 150 ml conical flask. For dispersion of the spores, fifty glass beads (4 mm in diameter) were added to the flask, and the flask was shaken on the incubator at 250 revolutions per minute (rpm) for 15 min. The obtained spore suspension was then diluted to make the spore concentration within the range 10^6^–10^8^ cells/mL. Ten-microliters of spore suspension was painted on the surface of the metal slide for ARTP mutagenesis. The mutagenic treatment distance was 2 mm, the processing power was 120 W, the flow rate of Helium was 10 standard liter per minute (slm), and the implantation time was 0–300 s. Following implantation, the samples were placed in electropolished (EP) tubes containing 1 ml normal saline, and then oscillated for 1 min. Following this, the spore suspension was diluted and painted on the isolation mediums. The strains were incubated at 45°C in an attempt to grow single colonies. Colony number was counted and the survival rate curve was plotted. The survival rate was calculated according to the following Eq. 1:

$$Survival\;rate\;\left(\%\right)=\frac{N_{C}}{N_{0}}\times 100$$ (1)

$N_{C}$ —— Colony number of the coated plate after ARTP implantation across different time points.

$N_{0}$ —— Colony number of the coated plate without ARTP implantation across different time points.

### Screening method for identification of thermostable mutants

Following implantation, dishes were incubated for 48 h at 65°C, and colonies that developed were defined as thermostable mutants. A single colony, characterized as displaying superior xylanase fermentation performance, was selected for the subsequent round of ARTP implantation and domestication at 70°C.

### Crude enzyme extraction method

Fermentation broth was centrifuged at 10,000 rpm at 4°C for 15 min. The supernatant was composed of crude enzyme solution, which was stored at 4°C.

### Genetic stability analysis

To assess the genetic stability of the thermostable mutant, the xylanase activities in the crude enzyme extraction of each generation were analyzed following five passages.

### Xylanase activity determination method

Xylanase activity was determined by the Somogyi Nelson method (Leite et al., 2016). 10% diluted xylanase was added to 1% beech xylan solution, and the ensuing reaction was carried out at a predetermined temperature for 10 min. Following this, dinitrosalicylic acid (DNS) was added and the mixture was incubated in a boiling water bath for 10 min. The absorbance at 540 nm was determined with an enzyme labeling instrument.

### Antioxidase activity determination method

Superoxide dismutase (SOD) activity was determined according to the xanthine oxidase method. Catalase (CAT) activity was determined by ammonium molybdate colorimetry. Peroxidase (POD) activity was determined according to the reaction of hydrogen peroxide catalyzed by POD. Polyphenol oxidase (PPO) activity was determined by the chromogenic reaction in which PPO catalyzed phenol to produce quinone. Antioxidant capacity (AOC) activity was determined according to the Fe^3+^ reduction reaction. All of the antioxidant enzyme activity determination kits were provided by the Nanjing Jiancheng Bioengineering Institute (Zhang et al., 2017).

### Optimum pH and pH stability

One percent substrate (beech xylan) solutions were prepared with different pH values (200 mmol/L Na_2_HPO_4_ -100 mmol/L citric acid buffer (for pH 4–8 buffer) and 50 mmol/L glycine-NaOH buffer (for pH 8.5–10 buffer)). Relative enzyme activities were calculated under different pH conditions with a maximum enzyme activity of 100% according to the above enzyme activity determination method. Xylanase was diluted with buffer (pH 4.0–11.0), respectively, and placed at 4°C for 36 h. Relative xylanase activities were measured relative to an untreated xylanase activity of 100%.

### Optimum reaction temperature and thermal stability

The substrate (beech xylan) solution was prepared with the optimal pH buffer, and the relative enzyme activities at different temperatures were measured according to the above enzyme activity determination method. Following this, the xylanases were placed in a 60–80°C water bath for 20 min, and then the enzyme activities were measured with the optimal pH buffer. The relative enzyme activities were calculated relative to an untreated xylanase activity of 100%.

## Results and discussion

### Mutagenic effect of ARTP on *M. thermophila*

#### Effect of ARTP mutagenic on the survival rate of *M. thermophila*

**FIGURE 1:**
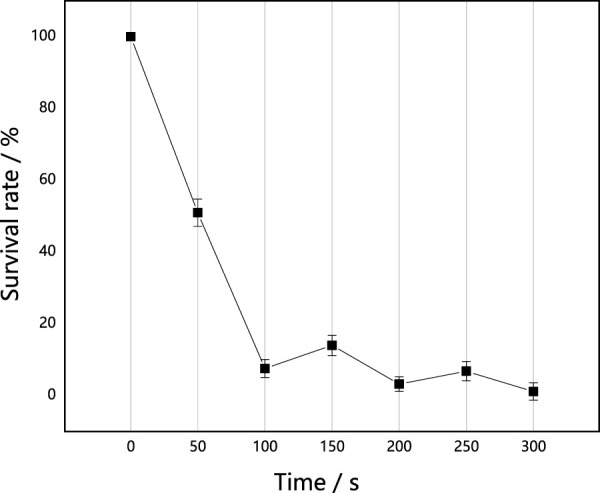
Effect of implantation time of ARTP on the survival rate of *M. thermophila*. Survival rate was the percentage of the number of colonies after implanted by ARTP to the none implanted. All experiments were carried out four times (biological duplicates and technical duplicates) and mean values presented with standard deviations.

As shown in Figure 1, the survival rate curve of *M. thermophila* resembled a “double saddle” shape following ARTP implantation, with two recovery peaks at the implantation times of 150 s and 250 s. Similar results were observed with low-energy ion implantation of *Bacillus. coagulans* (Yu et al., 2012) and *Trichoderma reesei* (Zhang and Jiang, 2011). For these, the “double saddle” survival curve was observed and was characteristics of the interaction between plasma and organisms. It was reminiscent of the classical “saddle” curve (Zhu, 2006), and not attributable to the specific effect produced by ion implantation into a certain microorganism. The appearance of “double saddle type” survival rate curves suggests that the mechanism of interaction between implanted ARTP and the microorganisms is complex, and it suggests that the mechanism of ion implantation mutation is likely different from traditional mutation methods. Previous studies (Zhang and Yu, 2008) have shown that survival rate curves are a reflection of the external performance of biological effects caused by implanted ions, while changes associated with antioxidases activities are reflective of various internal factors. The studies presented here thus focus on investigating the effects of ARTP on antioxidases activities of *M. thermophila*.

### Effects of ARTP mutagenesis on the activity of antioxidases in *M. thermophila*

#### Effect of ARTP implantation on SOD activity in *M. thermophila*

**FIGURE 2:**
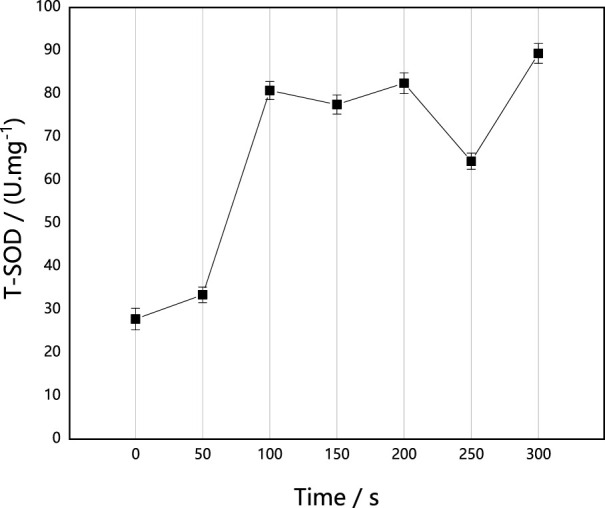
Effect of ARTP implantation time on T-SOD activities of *M. thermophila.* T-SOD activity was determined according to the xanthine oxidase method. Reagents preparation and results determination were carried out according to kit requirements. All experiments were carried out four times (biological duplicates and technical duplicates) and mean values presented with standard deviations.

SOD is an important member of the antioxidase system in organisms. It can catalyze the disproportionation of superoxide anion free radicals to produce oxygen and hydrogen peroxide, which play an important role in the balance of oxidation and antioxidation. It can resist and block the damage caused by oxygen free radicals to cells, repair damaged cells in time and recover the damage caused by oxygen free radicals. As shown in Figure 2, ARTP implantation induced the increase of SOD activity in *M. thermophila*. When the implantation time was 300 s, SOD activity increased by 221.13% as compared with the control, and it decreased under the conditions of 150 s and 250 s. In general, however, it was higher than that of untreated control strain. This may be attributed to active oxides, including the superoxide anion in ARTP, inducing *M. thermophila* to produce more SOD.

#### Effect of ARTP implantation on CAT and POD activities in *M. thermophila*

**FIGURE 3:**
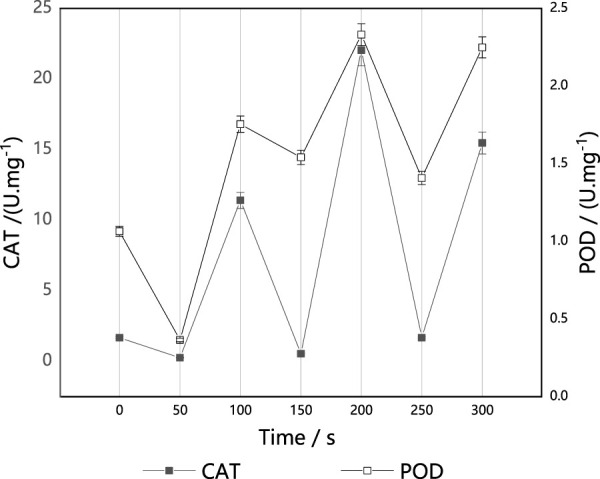
Effect of ARTP implantation time on CAT and POD activities of *M. thermophila*. CAT activity was determined by ammonium molybdate colorimetry. Reagents preparation and results determination were carried out according to kit requirements. All experiments were carried out four times (biological duplicates and technical duplicates) and mean values presented with standard deviations.

CAT and POD are enzymes that are able to scavenge H_2_O_2_ in cells. They play a key role in blocking free radical chain reactions. CAT directly catalyzes the decomposition of H_2_O_2_, while POD consumes H_2_O_2_ by catalyzing H_2_O_2_ to decompose other substrates. As shown in Figure 3, the activities of CAT and POD fluctuated with the increase of implantation time, contrary to the change in the trend of the survival curves. These results suggest that the increase of CAT and POD activities induced by ARTP are likely an adaptive response of *M. thermophila*. It is also likely a reason for the fluctuation in survival rate. In a short time (50 s), the implanted ions did not stimulate CAT and POD activities. With prolongation of the implantation time, CAT and POD activities were stimulated, and POD activity was higher than that of the untreated control strain. CAT activity was also much higher than that of the control strain at 100 s, 200 s and 300 s.

#### Effect of ARTP implantation on PPO activity in *M. thermophila*

**FIGURE 4:**
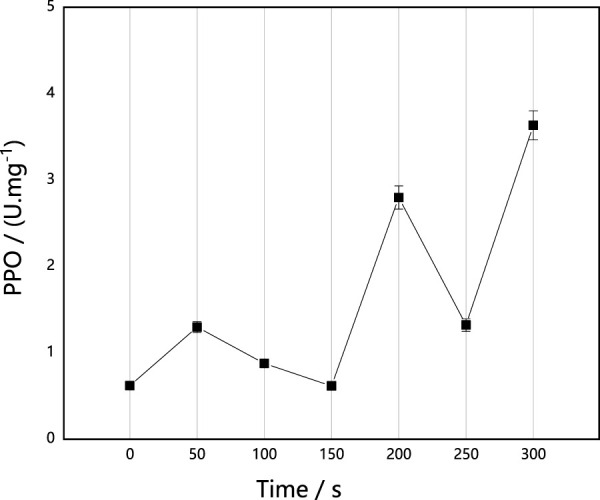
Effect of ARTP implantation time on PPO activities of *M. thermophila*. PPO activity was determined by the chromogenic reaction in which PPO catalyzed phenol to produce quinone. Reagents preparation and results determination were carried out according to kit requirements. All experiments were carried out four times (biological duplicates and technical duplicates) and mean values presented with standard deviations.

PPO is an oxidase that contains copper, which is able to oxidize monophenols and bisphenols to produce quinones. Quinones are able to inhibit and kill pathogenic microorganisms and contribute to resistance to disease. As shown in Figure 4, PPO activities were also activated after ARTP implantation in *M. thermophila*. Following implantation *via* ARTP, PPO activities were higher than those of the untreated control strain. In addition, PPO activity increased by 486.04% when the implantation time was 300 s. Further, the change in trend of PPO activity negatively correlated with the survival rate curve, suggesting that the change of PPO activity was also likely an adaptive response of *M. thermophila* under ARTP implantation.

#### Effect of ARTP implantation on AOC in *M. thermophila*

**FIGURE 5:**
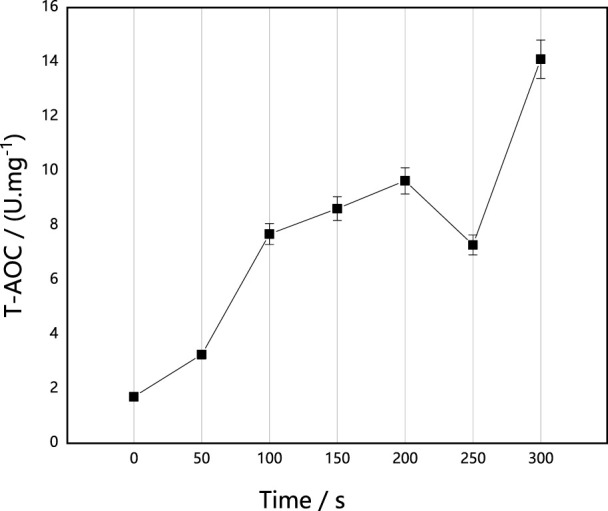
Effect of implantation time of ARTP on T-AOC of *M. thermophila*. T-AOC activity was determined according to the Fe^3+^ reduction reaction. Reagents preparation and results determination were carried out according to kit requirements. All experiments were carried out four times (biological duplicates and technical duplicates) and mean values presented with standard deviations.

AOC is composed of various antioxidant substances and antioxidant enzymes, such as vitamin C, vitamin E and carotene, and it can be used to evaluate the antioxidant capacity of bioactive substances. As shown in Figure 5, with the prolongation of implantation time, AOC activities were higher than those of the untreated control strain, and they generally increased, with the exception of a decrease at 250 s. Based on the effect of ARTP on survival rate and antioxidase activities of *M. thermophila*, we speculated that the enhancement of AOC activity may protect *M. thermophila* from the damage of free radicals and ROS caused by ARTP. In addition, the enhancement of AOC activity of *M. thermophila* could effectively resist oxidative stress after heat stress, which would effectively improve the heat resistance of *M. thermophila*. Therefore, the studies presented herein investigated the effects of ARTP implantation on the thermostability of *M. thermophila*.

#### Effects of ARTP implantation on the thermostability of *M. thermophila*

**FIGURE 6:**
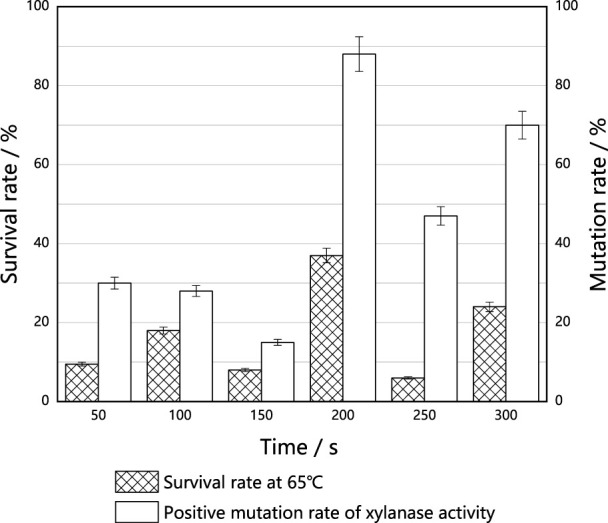
Effect of ARTP implantation time on the thermostable *of M. thermophila.* For *M. thermophila*, the optimum temperature was 60°C. On this basis, raised the culture temperature to 65°C. The effects of different ARTP implantation times on the survival rates of *M. thermophila were investigated*, and the xylanase activities of the surviving mutants were determined. The mutation rate was defined as the xylanase activity higher than 5% of the original strain. All experiments were carried out four times (biological duplicates and technical duplicates) and mean values presented with standard deviations.

Survival rates corresponding to each tested implantation time of ARTP are shown in Figure 6. The survival rate fluctuated with the increase in implantation time, and reached 37% at an implantation time of 200 s. Under this condition, the mutation rate associated with high production of xylase reached 88.0%. Antioxidase activities of *M. thermophila* also increased under this condition, suggesting that antioxidases play an important role in resisting cell death caused by heat stress. Subsequent mutagenesis screening experiments were also carried out at 200 s.

### Screening of thermostable xylanase-Producing strains

#### Screening spectrogram of the thermotolerant mutant

**FIGURE 7:**
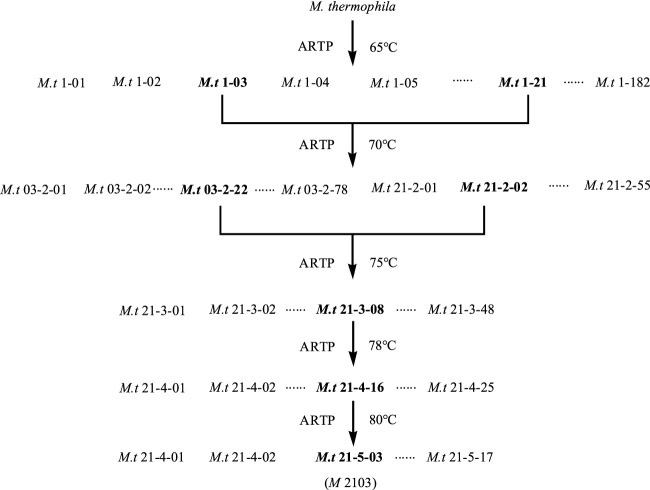
Screening spectrogram of the thermotolerant mutant *M* 2103. The first group number for the mutant strains was the serial number of the obtained mutant after the first mutagenesis by ARTP, the second group of number indicate the times of ARTP mutagenesis, and the third group of number was the serial number of the obtained mutant for this time of ARTP mutagenesis, and so on. The mutant strains with high xylanase activities after each mutagenesis were selected for the next mutation. After 5 mutagenesis, the culture temperature was gradually increased from 65°C to 80°C, and the thermostable mutant *M* 2013 with high activity of xylanase was finally obtained.

The mutation screening process is shown in Figure 7. In this study, following five ARTP mutagenesis treatments and increasing the culture temperature from 65°C to 80°C, one mutant strain, *M. thermophila* -2103 (*M* 2103), was obtained. Its xylanase activity increased from 1457.30 U/g to 1861.38 U/g and the activity remained stable after five generations. Results are shown in Table1.

**TABLE 1:** Stability of xylanase activity of *M* 2103 and *M.thermophila*.

Strains	Xylanase activities of every passages/(U·g^−1^)					
	*p*1	*p*2	*p*3	*p*4	*p*5	Averages
*M 2103*	1862.17	1860.62	1865.57	1860.25	1858.28	1861.38
*M.thermophila*	1433.87	1458.32	1438.06	1487.88	1468.37	1457.30

The stability experiments were carried out for five consecutive generations. All experiments were carried out four times (biological duplicates and technical duplicates) and mean values presented with standard deviations.

### Xylanase Properties of *M* 2103 and the original strain *M.thermophila*

#### Optimum reaction pH and pH stability

**FIGURE 8:**
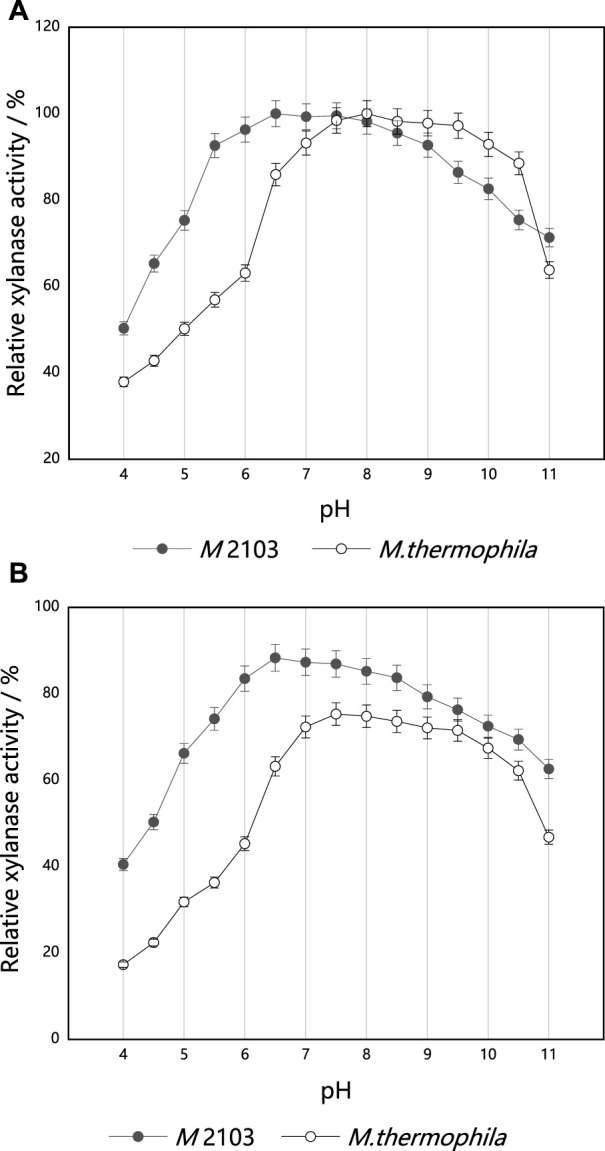
Optimum reaction pH and pH stability of xylanase from mutant *M* 2103 and *M. thermophila*; **(A)** The optimum pH of xylanase from mutant *M* 2103 and *M. thermophila*; **(B)** pH stability of xylanase from mutant *M* 2103 and *M. thermophila*. All experiments were carried out four times (biological duplicates and technical duplicates) and mean values presented with standard deviations.

The effects of different pH values on xylanase activities are shown in Figure 8A. The mutant *M* 2103 displayed significantly higher relative xylanase activities than the original strain in the acidic (pH 4.0–7.0) range. In the pH range of 6.0–8.5, the xylanase activities were relatively stable, with an optimal pH of 6.5. When the pH was higher than 8.5, xylanase activities began to decrease. When the pH was 10.0, the remaining xylanase activity was 82.63%. The fermentation crude enzyme solution of mutant *M* 2103 was diluted appropriately with buffer solution of pH 4.0–11.0, respectively. After being placed at 4°C for 36 h, the xylanase activity of *M* 2103 was compared with that of the untreated crude enzyme solution (Figure 8B). At a pH of 11.0, the xylanase activity of *M* 2103 could be maintained above 65%. Compared with the alkaline condition, the xylanase activity of *M* 2103 in the acidic range was higher and relatively stable.

#### Optimum reaction temperature and temperature stability

**FIGURE 9:**
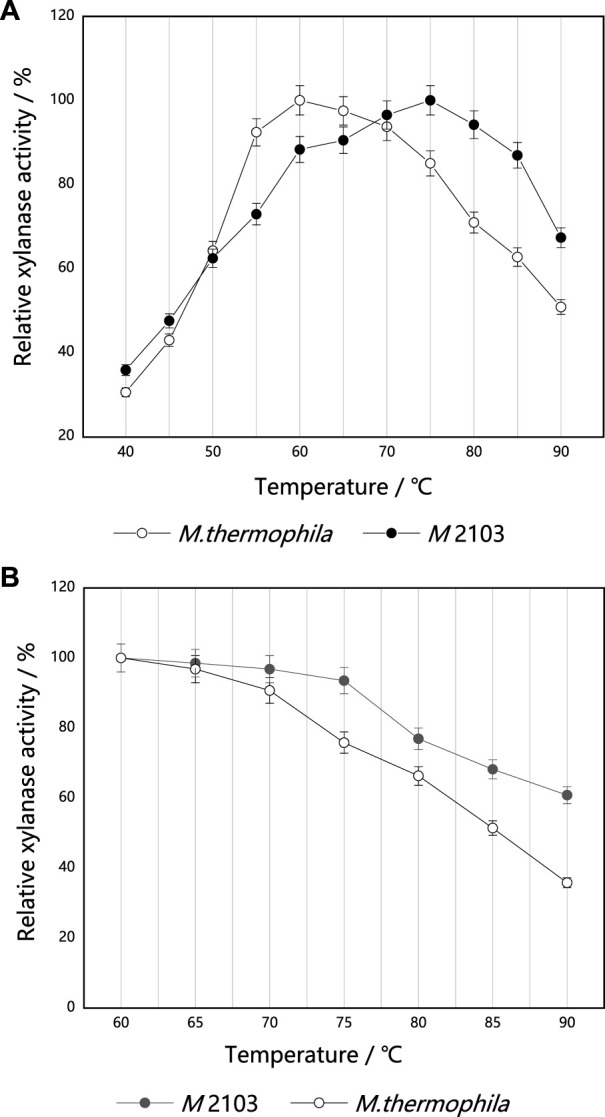
Optimum reaction temperature and thermal stability of xylanase from mutant *M* 2103 and *M. thermophila*. **(A)** The optimal reaction temperature of xylanase from mutant *M* 2103 and *M. thermophila* **(B)** Thermal stability of the xylanase from mutant *M* 2103 and *M. thermophila.* All experiments were carried out four times (biological duplicates and technical duplicates) and mean values presented with standard deviations.

The optimum reaction temperature of the original strain *M. thermophila* and mutant *M* 2103 were measured under their corresponding optimum reaction pH (7.5 and 6.5) (Figure 9A). The optimum reaction temperature for *M. thermophila* to produce xylanase was determined to be 60°C, and the optimum reaction temperature for *M* 2103 was 75°C (it remained stable in the range of 70°C–85°C). Its residual xylanase activity was 67% at 90°C. The thermal stability of the original strain *M. thermophila* and mutant *M* 2103 were then determined. As shown in Figure 9B, the residual activity of *M. thermophila* decreased to about 90% after being treated at 70°C for 20 min, while the activity of *M* 2103 was almost unchanged. After being treated at 90°C for 20 min, the residual activity of *M. thermophila* decreased to about 35%, while *M* 2103 had activity of around 60%. These results suggest that the thermal stability of *M* 2103 was superior to that of *M. thermophila.*

## Conclusion

A thermostable mutant, *M* 2103, with high xylanase productivity was obtained by ARTP mutagenesis. Compared with the original strain, the xylanase activity increased by 21.71%, the optimum temperature reached 75°C from 60°C, and its reaction temperature for xylanase production remained stable in the range of 70°C–85°C. *M* 2103 displayed a significantly higher relative xylanase activity than the original strain in the acidic (pH 4.0–7.0) range, and the xylanase activity was relatively stable in the pH range of 6.0–8.5. In addition, ARTP mutagenesis can stimulate antioxidase system (SOD, CAT, POD, PPO, and AOC) activities in *M. thermophile*, which SOD activity increased by 221.13%, and PPO activity increased by 486.04%. These studies showed that the increasing of antioxidase system (SOD, CAT, POD, PPO, and AOC) activities in *M. thermophile* reduced the oxidative damage caused by ARTP, and helped to improve its heat resistance.

In this study, ARTP was used to mutate *M. thermophile*, and improved its thermostable and xylanase activity. This method has the advantages of low jet temperature, uniform plasma production, no vacuum device, simple operation, low cost, and obvious interaction with biological macromolecules and cells compared with traditional mutagenesis methods. This study provide an alternative biocatalyst for the production of xylooligosaccharide, and it has potential application in the fuel ethanol industry.

## Data Availability

The raw data supporting the conclusion of this article will be made available by the authors, without undue reservation.
